# Development of an *in vitro* media perfusion model of *Leishmania major* macrophage infection

**DOI:** 10.1371/journal.pone.0219985

**Published:** 2019-07-24

**Authors:** Alec O’Keeffe, Lauren Hyndman, Sean McGinty, Alaa Riezk, Sudaxshina Murdan, Simon L. Croft

**Affiliations:** 1 Department of Infection and Immunology, London School of Hygiene and Tropical Medicine, London, United Kingdom; 2 Department of Pharmaceutics, UCL School of Pharmacy, University College London, London, United Kingdom; 3 Division of Biomedical Engineering, University of Glasgow, Glasgow, United Kingdom; Taibah University, SAUDI ARABIA

## Abstract

**Background:**

*In vitro* assays are widely used in studies on pathogen infectivity, immune responses, drug and vaccine discovery. However, most *in vitro* assays display significant differences to the *in vivo* situation and limited predictive properties. We applied medium perfusion methods to mimic interstitial fluid flow to establish a novel infection model of *Leishmania* parasites.

**Methods:**

*Leishmania major* infection of mouse peritoneal macrophages was studied within the Quasi Vivo QV900 macro-perfusion system. Under a constant flow of culture media at a rate of 360μl/min, *L*. *major* infected macrophages were cultured either at the base of a perfusion chamber or raised on 9mm high inserts. Mathematical and computational modelling was conducted to estimate medium flow speed, shear stress and oxygen concentration. The effects of medium flow on infection rate, intracellular amastigote division, macrophage phagocytosis and macropinocytosis were measured.

**Results:**

Mean fluid speeds at the macrophage cell surface were estimated to be 1.45 x 10^−9^ m/s and 1.23 x 10^−7^ m/s for cells at the base of the chamber and cells on an insert, respectively. *L*. *major* macrophage infection was significantly reduced under both media perfusion conditions compared to cells maintained under static conditions; a 85±3% infection rate of macrophages at 72 hours in static cultures compared to 62±5% for cultures under slow medium flow and 55±3% under fast medium flow. Media perfusion also decreased amastigote replication and both macrophage phagocytosis (by 44±4% under slow flow and 57±5% under fast flow compared with the static condition) and macropinocytosis (by 40±4% under slow flow and 62±5% under fast flow compared with the static condition) as measured by uptake of latex beads and pHrodo Red dextran.

**Conclusions:**

Perfusion of culture medium in an *in vitro L*. *major* macrophage infection model (simulating *in vivo* lymphatic flow) reduced the infection rate of macrophages, the replication of the intracellular parasite, macrophage phagocytosis and macropinocytosis with greater reductions achieved under faster flow speeds.

## Introduction

Traditional cell culture methods typically rely on either immortalized cell lines or primary isolated cells grown in designed nutritious media on non-physiological substrates, such as functionalized plastic and glass. Although these methods have been at the core of *in vitro* studies on many basic biological processes, they provide a limited platform owing to both their inadequate representation of key physiological characteristics and their relevance to disease models [1]. One area that is often overlooked in cell culture models is the transport and movement of nutrients around cells, which occurs through fluid flow in the body. This could impact on the growth and survival of pathogens in intracellular models as infection is reliant on nutrients provided by the host cell and cell-cell interactions. Within the mammalian body, rates of fluid flow vary from the rapid plasma flow of 9.8 ml/min in the portal vein of the rat [2] to the slower 0.19 μl/min rate of interstitial fluid drainage from rat brains [3]. Interstitial fluid in tissues, including skin, arises from the normal leakage of plasma from blood vessels and has a composition that is similar to that of blood plasma [4]. It is estimated that up to 20% of the body’s mass is made up of interstitial fluid [4].

Leishmaniasis is an infectious disease caused by protozoa parasites of the genus *Leishmania*, which have two distinct life cycle stages: an extracellular motile promastigote form in the sandfly vector and an intracellular amastigote form that survives and multiplies in the phagolysosomal compartment of mammalian macrophages [5]. Two predominant forms of the disease result from infection by *Leishmania* parasites, the potentially fatal visceral leishmaniasis (VL) and the self-curing, but disfiguring, cutaneous leishmaniasis (CL). Although macrophages of the liver and spleen infected with *Leishmania donovani*, the cause of VL, are exposed to plasma flow rates, in the skin sites of infection in CL, infected macrophages are exposed to interstitial fluid. While the exact speed of interstitial fluid flow through the CL lesion is not known, measurements have shown that interstitial fluid flow in uninfected human skin is of the order of 0.1–2 μm/s [4,6,7]. Most *in vitro* studies on invasion, infection, immunology and drug discovery within the *Leishmania* field have so far been performed using macrophages grown under static culture conditions [8–10]. To simulate some of the more complex interactions between the parasite and macrophages in the host we selected the Quasi Vivo 900 media perfusion system (QV900) with a 6-chamber optical tray, to enable the imaging of cultures *in situ* at a flow rate similar to that of interstitial fluid. Here we describe the effect of media perfusion on the infection of mouse peritoneal macrophages with *Leishmania major* and use mathematical modelling to estimate the flow speed, shear stress and oxygen tension at the host cell surface. In addition, we have determined the impact of flow on intracellular amastigote division, and host cell phagocytosis and macropinocytosis.

## Material and methods

### Media perfusion system

Quasi Vivo media perfusion systems (Kirkstall Ltd, Rotherham, UK) were selected as they enable the direct observation of infected cells exposed to different medium perfusion rates and the continuous monitoring of infection. The Quasi Vivo systems include the QV500, an individual chamber system, and the QV900, a six chamber optical tray which permits connecting of chambers in series. We selected the QV900 given that it is more suited to high-throughput testing. Mathematical and computational modelling of the QV500 [11] has shown that the speed of media at the surface of cells cultured at the base of the chambers is within the range of interstitial fluid flow rates [4,6,7] in humans for a flow rate of 360μl/min. However, the QV900 chambers differ in geometry and in particular are significantly deeper, having a depth of 22 mm compared with 12 mm in the QV500. As a result of this difference in chamber geometry, the fluid environment in the QV900 is markedly different from that in the QV500 at the same flow rate. Therefore, we inserted a 3D printed block composed of Nylon 12 (Kirkstall Ltd) in selected chambers to enable us to study cells cultured at different depths in the QV900 chamber. Mathematical and computational modelling (see sections below) were utilized to calculate the insert height that would ensure the cell surface flow speeds would fall within the reported range for interstitial flow in the skin. All six chambers of the QV900 were connected in series with the last three of the chambers containing inserts. A peristaltic pump (Parker Hannifin, UK), external to the CO_2_ incubator, continuously circulated culture media through the system.

### Modelling fluid flow and oxygen transport in the QV900 system

**Fig 1:**
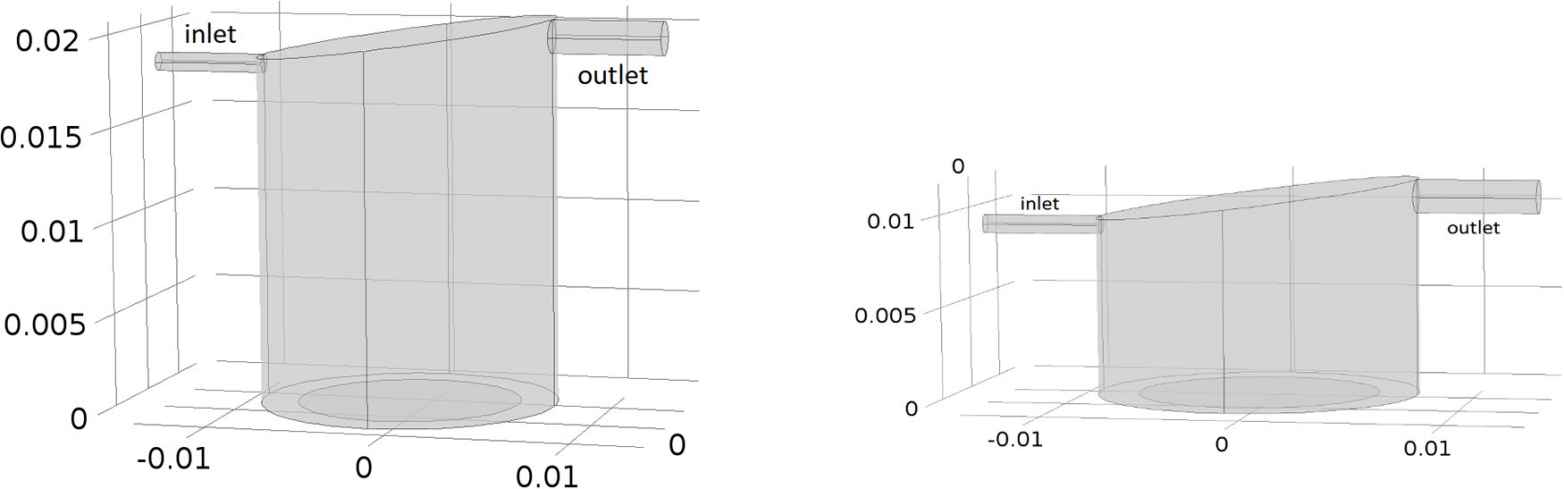
Left: Idealized 3D geometry of a single QV900 chamber. This represents the computational domain for the case where the cells are placed at the base of the chamber. Right: Computational domain for cells placed on a 9mm insert. Note that length scales are in m.

COMSOL Multiphysics, a commercially available finite element analysis software, was used to perform simulations in this study. Initial modelling focused on single chamber studies to establish the size of the insert required to achieve the desired cell surface flow speeds. Subsequently, simulations were conducted for six chambers connected in series, matching the experiments. Fig 1 illustrates the computational domains for cells placed at the base of the chamber (left) and on a 9mm insert (right). Note that in both cases the chambers are identical in dimensions, but since there is assumed to be no fluid flow beneath the insert, the depth of this computational domain is reduced.

The fluid flow was modelled using the Navier-Stokes equations, assuming that the media is an incompressible Newtonian fluid. The transport of oxygen throughout the media was modelled by convection and diffusion. The cells were assumed to reside at the base of each computational domain on circular coverslips of diameter 12mm. Oxygen consumption by the cells was described using Michaelis-Menten kinetics and implemented through a flux boundary condition. The equations and parameter values used in the simulations are detailed in the supplementary material (S1 File).

### Culture systems

#### *Leishmania* parasites

*L*. *major* (MHOM/SA/85/JISH118) amastigotes were obtained and isolated from mouse skin lesions. They were allowed to transform to promastigotes and were maintained in Schneider’s insect medium (Sigma Aldrich, UK) supplemented with 10% heat inactivated foetal calf serum (HiFCS) (Harlan, UK) at 26°C. The parasites were routinely passaged through BALB/c mice (Charles River, UK) and low passage number promastigotes (< passage number 3) were used for experiments as infectivity has been shown to decrease with time of parasite cultivation [12].

All animal experiments were conducted under license (project license 70/6997 or X20014A54) in accordance with UK Home Office approval, EU regulations, EU directive 2010/63/EU. Protocols followed in these studies for the isolation of peritoneal macrophages was approved by the LSHTM Animal Welfare and Ethics Review Board. The mice are housed in green line I.V.C.s, 5 mice per cage, with access to food and water ad libitum. At all stages the 3Rs (replacement, reduction and refinement) were taken into consideration.

#### Macrophages

Mouse peritoneal macrophages (PEM) were isolated from CD-1 mice (Charles River, Margate, UK) by abdominal lavage [13] with RPMI-1640 medium containing 1% penicillin and 1% streptomycin (Sigma, UK).

THP1 cells (ATCC TIB-202, UK) were maintained in RPMI-1640 containing 10% HiFCS (Harlan, UK) and passaged by a 1:10 split weekly.

#### Infection of macrophages by *L*. *major* promastigotes

Macrophages were plated on 12mm round glass coverslips (Bellco, US) placed in 24 well plates (Corning, UK) at a density of 4 x 10^5^ cells per well in RPMI-1640 media supplemented with 10% HiFCS. The plates were incubated at 37°C in 5% CO_2_ for 24 hours. *L*. *major* stationary phase promastigotes were counted and dilutions of different concentrations of parasite (2 x 10^5^ to 6 x 10^7^) were pre-prepared in media to give initial parasite: macrophage ratios within the range of 0.5:1–15:; promastigotes were added to the macrophage cultures. The plates were placed in an incubator maintained at 34°C (temperature relevant for CL [14]) and 5% CO_2_ for 24 hours. Subsequently, two thirds of the glass coverslips were transferred to the media perfusion system and maintained under flow conditions at a flow speed of 360 μl/min for 72 hours in a 34°C, 5% CO_2_ incubator. The remaining coverslips were used for the static control, with macrophages maintained in the same culture medium without flow. The cells were methanol (Sigma, UK) fixed and stained with Giemsa’s stain (Sigma, UK). The infection rate of the macrophages was assessed visually using an oil immersion microscope (100x magnification Zeiss, UK) by counting the number of infected cells per 100 macrophages. Values for percentage infection throughout are shown as mean ± standard deviation.

#### Incorporation of 5-ethynyl-2'-deoxyuridine (EdU) into dividing amastigotes

Invitrogen Click-iT EdU Imaging Kit (Invitrogen, UK) was used to measure 5-ethynyl-2´-deoxyuridine incorporation as a measure of proliferation. Only the dividing parasites should incorporate the EdU as the macrophage populations used are fully differentiated non dividing cells. The kit comprised of a fluorescently labelled DNA base, which is incorporated into DNA synthesized during amastigote division. Experiments, based on the methodology of Tegazzini et al. [9], were conducted as before except that PEMs were infected with a ratio of 3 *L*. *major* promastigotes: 1 macrophage and maintained at 34°C, 5% CO_2_ in an incubator for 24 hours. Media used contained 50 μM EdU. After 24 hours, cells were placed in a new 24-well plate and were fixed in 4% Paraformaldehyde (PFA) (Sigma, UK) for 15 minutes at room temperature. The samples were treated with 0.2% Triton X-100 (Sigma, UK) in PBS (Sigma, UK) for 20 minutes and then 1% BSA (Sigma, UK) in PBS for 10 minutes. Click-iT reaction cocktail was prepared according to instructions in Invitrogen Click-iT EdU Imaging Kit. Click-iT reaction cocktail (0.5 mL) was added to each well containing a coverslip, and plates were incubated for 30 minutes at room temperature, protected from light. Cells were then washed with 1 mL of 3% BSA in PBS, then incubated with 300 mM DAPI stain (Sigma, UK) for 10 minutes to stain the nucleus of the cell, coverslips were mounted onto slides and imaged using a confocal microscope (Zeiss LSM510 Axiovert, Germany). The lasers used were Laser Diode: 405 nm for DAPI excitation and Argon laser: 458, 488, 514 nm for EdU excitation. Images were captured at 40x magnification and analysed using Velocity software (PerkinElmer, US) to automatically count the total number of nuclei in each field of view and this is proportional to the total cell number. A minimum of 100 macrophages were counted microscopically from each coverslip. Images were manually viewed to count the number of fluorescent and non-fluorescent parasites within each cell. The results were exported and analysed with Graphpad Prism.

#### Measurement of macrophage functions

Phagocytosis by macrophages was initially evaluated using 0.5,1 and 2 μm diameter fluorescent red labelled latex beads (carboxylate-modified polystyrene) (Sigma-Aldrich, UK) [15,16]; 2 μm beads were eventually selected as they showed maximal signal. Macrophages were infected with parasites, then transferred to the three flow conditions as described above. To each well, 2μm beads (9.12 x 10^7^ latex beads/ml) were added and the cells were incubated for 0.5, 1, 2, 4 and 24 hours at 34 °C under the three different flow conditions. The experiment was terminated by washing the cells 4 times with ice-cold PBS pH 7.4 to remove non-internalized latex beads, followed by the addition of 1 ml of 0.5% Triton X-100 in 0.2 M NaOH to lyse the cells. Phagocytosis was quantified by the analysis of the cell lysate using a fluorescence plate reader (Spectramax M3, at excitation and emission wavelengths set at 575 and 610 nm), calibrated with standard solutions containing different amount of latex beads in a cell lysate mixture. Uptake was expressed as the number of latex beads associated per mg of cellular protein, the protein content of the cell lysate being measured using a Micro BCA protein kit (Thermo Fisher, UK) assay as per supplier’s instructions. For control studies, 1 μg/ml cytochalasin D was used as a phagocytosis inhibitor (Sigma-Aldrich, UK) by incubation with macrophages for 2 hours prior to addition of the latex beads. Phagocytosis was completely inhibited after 0.5, 1, 2 and 4 hours of incubation with cytochalasin D and 90% after 24 hours.

#### Macropinocytosis

Macropinocytosis was measured using a fluorescence-labeled dextran dye [17] (pHrodo Red dextran, average molecular weight of dextran 10,000 MW, Thermo Fisher, UK). This dye has a pH-sensitive fluorescence emission that increases in intensity with increasing acidity while exhibiting minimal fluorescence at neutral pH. Macrophages were infected with parasites and then transferred to the three flow conditions as described above. Macrophages were washed 3 x by Live Cell Imaging Solution (Thermofisher, UK) and the cells were returned to RPMI 1640 + 10% hiFCS containing 40 μg/mL pHrodo Red dextran (1 ml for each well) and incubated at 34 °C / 5% CO_2_ for 0.5, 1, 2, 4 and 24 hours under the three different flow conditions. At each time point, the cells were washed with Live Cell Imaging Solution and macropinocytosis was analysed by a Spectramax M3 at excitation and emission wavelengths set at 560 and 585 nm respectively. Chlorpromazine hydrochloride 10 μg/ml, a known inhibitor (Sigma-Aldrich, UK), was used as a control and was incubated with macrophages for 2 hours prior to addition of fluorescence-labeled dextran dye. Macropinocytosis was completely inhibited after 0.5, 1, 2 and 4 hours of incubation with chlorpromazine hydrochloride and by 90% after 24 hours.

## Results

### Establishment of infected macrophages in Quasi Vivo systems

Initial experiments using the Quasi Vivo systems involved the adaptation of the QV900 for our experimental purposes and the establishment of media perfusion within the system with a focus on the optimization of conditions to maintain viable cells within the system. A second objective was to ensure that an infection with *Leishmania* parasites could be sustained, as shown in subsequent experiments.

### QV900 media perfusion system modelling

Initially, single chamber simulations were carried out to estimate the height of the insert required to ensure the cell surface flow speed would be within the reported range for interstitial flow in the skin. Table 1 shows the estimated speed of the culture medium on the cell surface for various insert heights. It is clear that a 9mm insert would enable a culture medium flow speed in line with the speed of interstitial fluid flow in the skin, and therefore this height was chosen for subsequent modelling and experiments.

**Table 1:** Simulation results show that a 9mm insert is required to bring the cell surface flow rate in line with the values of 0.1–2 μm/s reported in [4,6,7].

Insert height (mm)	Mean cell surface flow speed (m/s)
0mm	1.33 x 10^−9^
5mm	1.80 x 10^−8^
6mm	3.05 x 10^−8^
7mm	5.01 x 10^−8^
8mm	7.77 x 10^−8^
9mm	1.17 x 10^−7^

**Fig 2:**
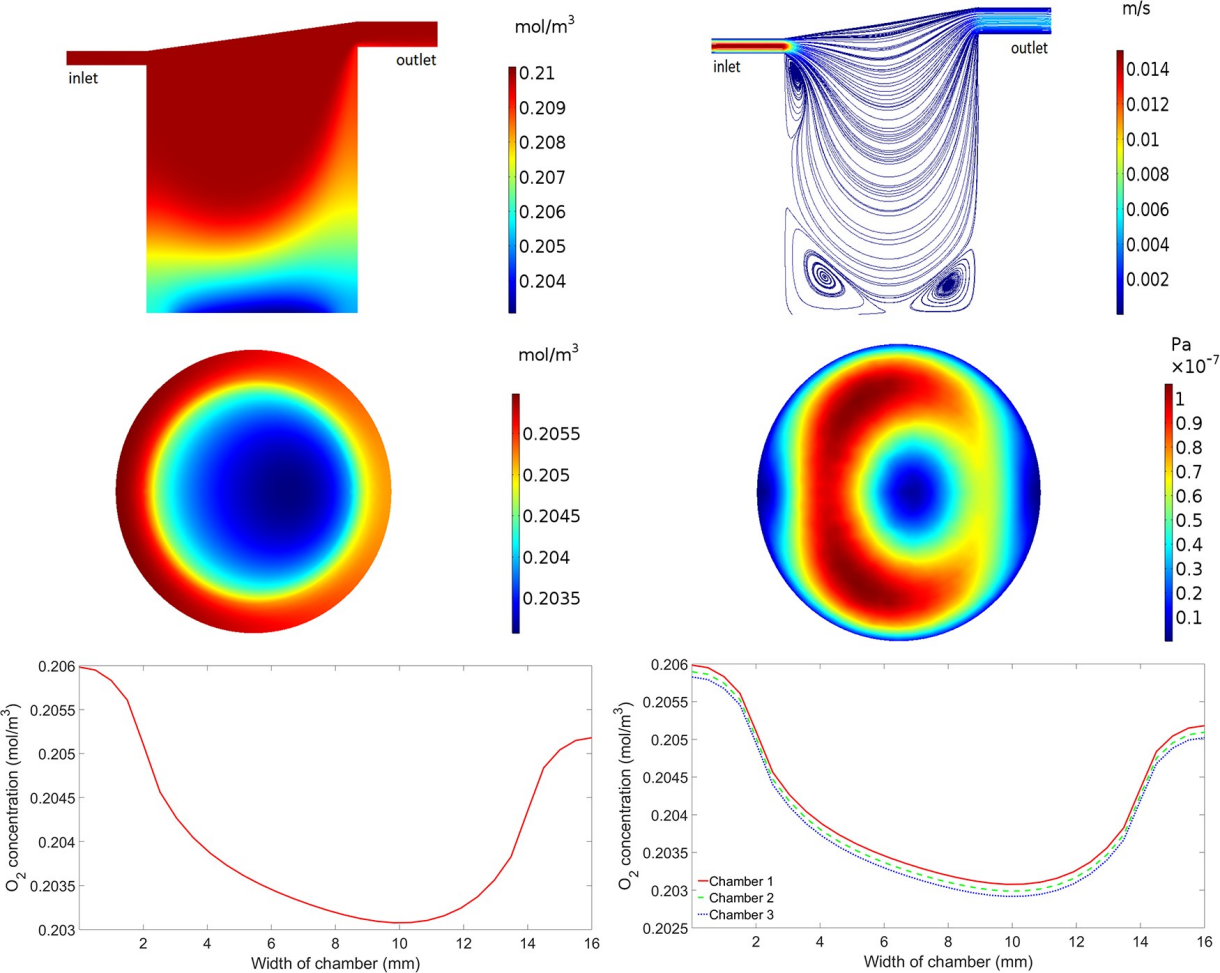
Simulation results for cells at the base of the chamber. Upper left: Oxygen concentration in chamber 1. Upper right: Flow profile in chamber 1. Middle left: Oxygen concentration at the base of chamber 1. Middle right: Magnitude of the shear stress at the base of chamber 1. Lower left: Oxygen concentration across the centre of the base of chamber 1. Lower right: Oxygen concentration across the centre of the base of chambers 1, 2 and 3.

**Fig 3:**
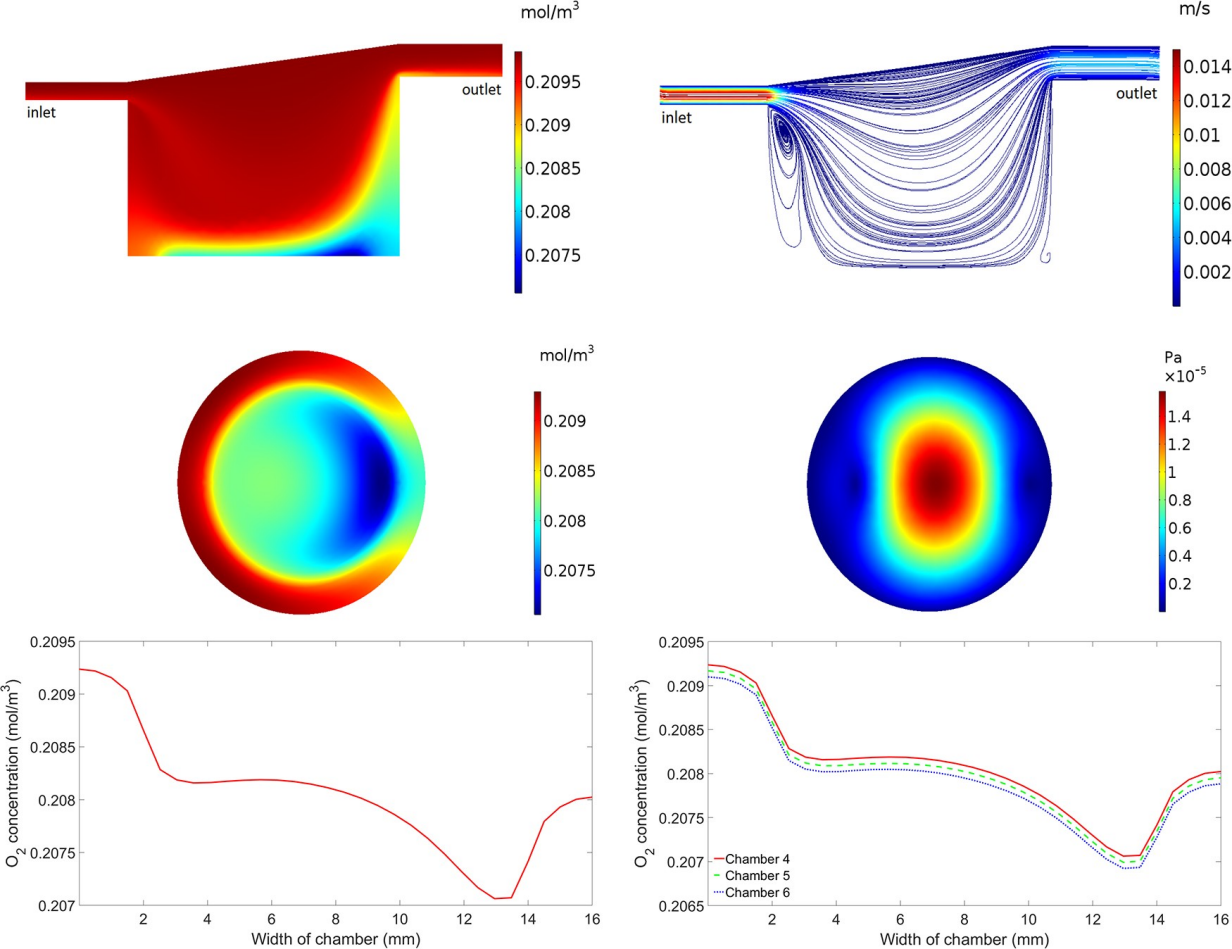
Simulation results for cells on top of the 9mm insert. Upper left: Oxygen concentration in chamber 4. Upper right: Flow profile in chamber 4. Middle left: Oxygen concentration at the base of chamber 4. Middle right: Magnitude of the shear stress at the base of chamber 4. Lower left: Oxygen concentration across the centre of the base of chamber 4. Lower right: Oxygen concentration across the centre of the base of chambers 4, 5 and 6.

Subsequent mathematical and computational modelling was carried out to match the experimental set up, i.e. we simulated six chambers connected in series, with the first three chambers having cells residing at the base and the next three chambers having cells raised 9mm. Fig 2 illustrates results which are representative of the first three chambers in the series. All plots in Fig 3 show the results for chamber one, with the exception of the lower right plot which shows results for the first three connected chambers. The simulated oxygen concentration decreases from the inlet of the chamber, where oxygen is supplied, to the base of the chamber, where oxygen is consumed by the cells (Fig 2, upper left). At the base of the chamber, the oxygen concentration is highest at the inlet side, reducing towards the centre, before rising again at the outlet side of the chamber (Fig 2, middle left). This gradient, clearly highlighted in the lower left plot of Fig 2, is a combined result of the complex flow field and the fact that oxygen consumption only occurs on the part of the base where the cells reside. Similar results for chambers two and three are shown in the lower right plot of Fig 2. The oxygen concentrations are lower in each consecutive chamber as a result of consumption, but the pattern of oxygen concentrations across the base is consistent between each chamber. The overall gradient of oxygen at the base of the first three connected chambers ranges from a maximum of 0.2059 mol/m^3^ in chamber one to a minimum of 0.2029 mol/m^3^ in chamber three.

The upper right plot of Fig 2 illustrates the flow speed and streamlines (the trajectories that particles would follow), demonstrating how the media flows through the chamber. The media flow is fastest at the inlet and outlet, and flow recirculation zones are observed beneath the inlet and at the base of the chamber. In these areas, the media is recirculated which could result in parasites and oxygen/drug molecules being trapped. The flow speed of the media at the base of the first chamber is consistent with the second and third chambers and has a mean value of 1.45 x 10^−9^ m/s. We note that this is slightly higher than the mean flow speed obtained in a single chamber (Table 1) as a result of the altered fluid dynamics due to connecting the chambers in series. A 2D representation of the magnitude of the shear stress the cells are under at the base of the chamber is shown in the middle right plot of Fig 2. The shear stress values range from a minimum of 2.09 x 10^−10^ Pa to a maximum of 1.06 x 10^−7^ Pa which is consistent with the second and third chambers.

Fig 3 illustrates results which are representative of the last three chambers in the series i.e. where the cells are placed on a 9mm insert. All plots show the results for chamber four (the first chamber in the series which has the cells raised by 9mm), with the exception of the lower right plot which shows results for the last three connected chambers (chambers 4, 5 and 6). The inclusion of the 9mm insert has an impact on both the pattern and magnitude of the oxygen concentration and fluid flow. Higher oxygen concentrations are observed throughout the whole chamber when compared to the chambers without an insert (Fig 3, upper left), and the minimum oxygen concentration at the base of the chamber occurs closer to the outlet side than when compared to the chambers without an insert (Fig 3, middle left). The oxygen concentration gradient across the base of the chamber is clearly highlighted in the lower left plot of Fig 3. Again, this pattern is a combined result of the complex flow field and the fact that oxygen consumption only occurs on the part of the base where the cells reside. Similar results for chambers 5 and 6 are shown in the lower right plot of Fig 3. As before, the oxygen concentration decreases between consecutive chambers due to consumption but the pattern remains the same. The overall gradient of oxygen at the base of the last three connected chambers ranges from a maximum of 0.2093 mol/m^3^ in chamber 4 to a minimum of 0.2069 mol/m^3^ in chamber 6.

The depth of the last three chambers in the series is dramatically reduced due to the 9mm insert which has a large impact on the pattern of flow (Fig 3, upper right). In this case, the only flow recirculation zone is observed beneath the inlet to the chamber. The mean flow speed of the media at the cells on top of the insert in the fourth chamber is 1.23 x 10^−7^ m/s–two orders of magnitude higher than in the chambers without the insert. This is consistent with the fifth and sixth chambers where the mean flow speed is also 1.23 x 10^−7^ m/s. Due to the difference in the flow profile, the pattern of shear stress at the base of the chamber is also noticeably different when compared to the chambers without an insert (Fig 3, middle right). The shear stress values range from a minimum of 5.75 x 10^−9^ Pa at the edges of the base of the chamber to a maximum of 1.58 x 10^−5^ Pa at the centre of the base of the chamber. This is again consistent with the fifth and sixth chambers.

### Determination of optimal experimental conditions

**Fig 4:**
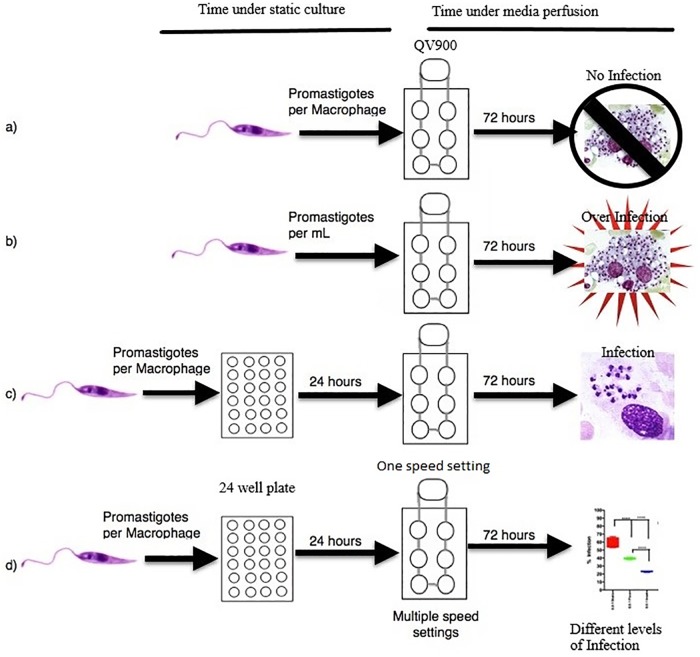
Schematic overview of the initial infection experiments.

**Fig 5:**
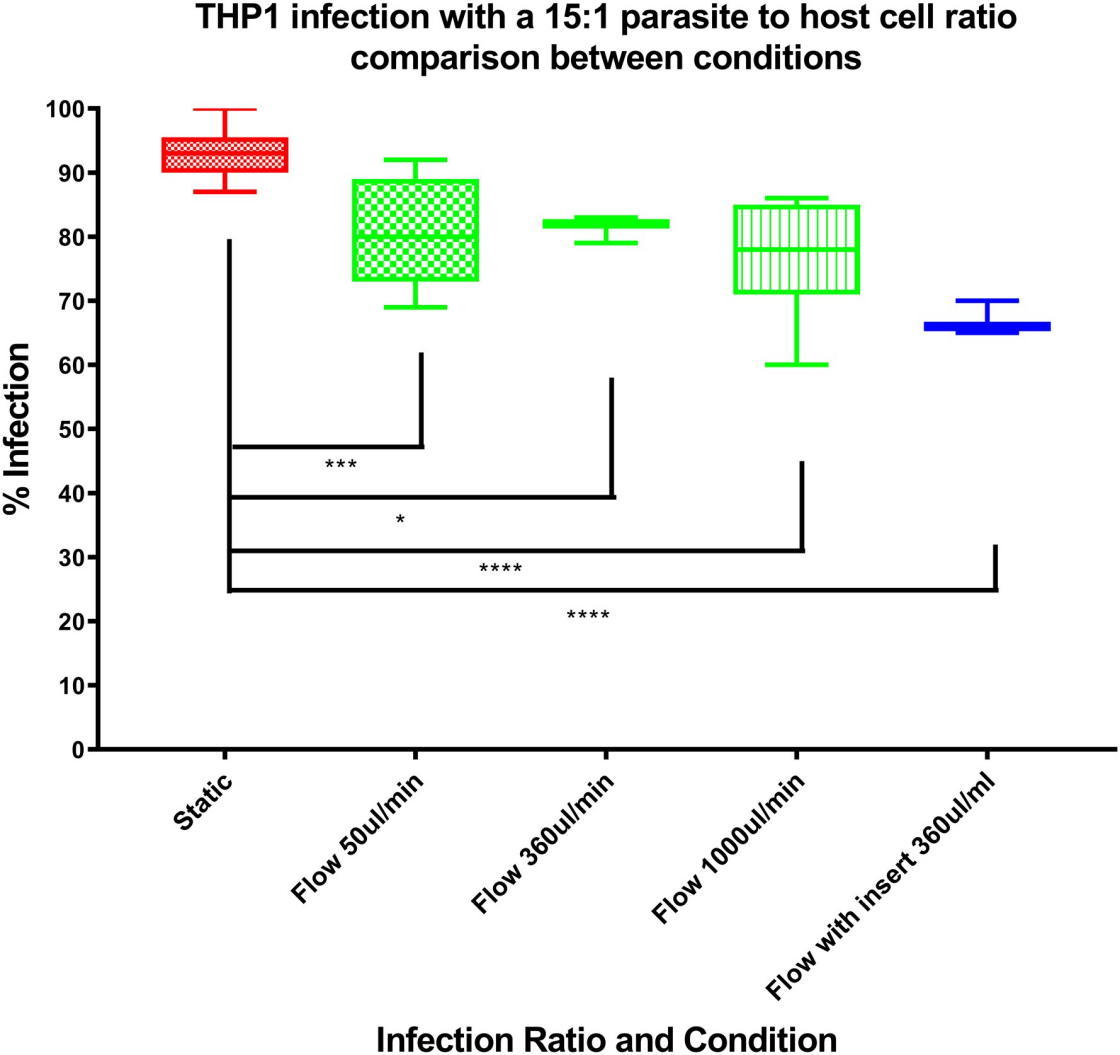
Influence of flow rate on percentage infection at a *L*. *major* promastigote to macrophage ratio of 15:1.

Initially, we used both THP1 cells and PEMs at different concentrations from 1 x 10^5^ to 4 x 10^5^ cells per chamber, to establish a viable, reproducible and measurable system. After preliminary work using THP1 cells PEMs were selected for further studies as in this macrophage type infections with *L*. *major* were easier to establish and to sustain. Peritoneal macrophages at 4 x 10^5^ cells per well were chosen as this concentration gave the most reproducible results following a series of studies at different conditions that were investigated (Fig 4). The initial studies showed that:

1. the addition of parasites in the medium during perfusion, at parasite: macrophage ratios from 0.5:1 to 10:1, resulted in zero macrophage infection after 72 hours and was therefore not pursued.
2. a set number of parasites per ml of circulating media from 4 x 10^5^ to 1.2 x 10^6^ cells per ml caused the parasites to collect within the chambers resulting in over-infection and bursting of the macrophages at the 72 hour time point. This approach was also not pursued.
3. the addition of different numbers of promastigotes before the initiation of media perfusion at parasite:macrophage ratios from 0.5:1 to 25:1 in the medium for a 24 hour pre-infection before media perfusion resulted in a controllable, reproducible infection after 72 hours. This approach was adopted.
4. there were decreased rates of macrophage infection with increasing flow rates from 50 to 360 to 1000μl/min (Fig 5). A flow rate of 360 μl/min was subsequently selected as it gave an acceptable level of infection.

### Infection of mouse peritoneal macrophages (PEMs) in the media perfusion system

**Fig 6:**
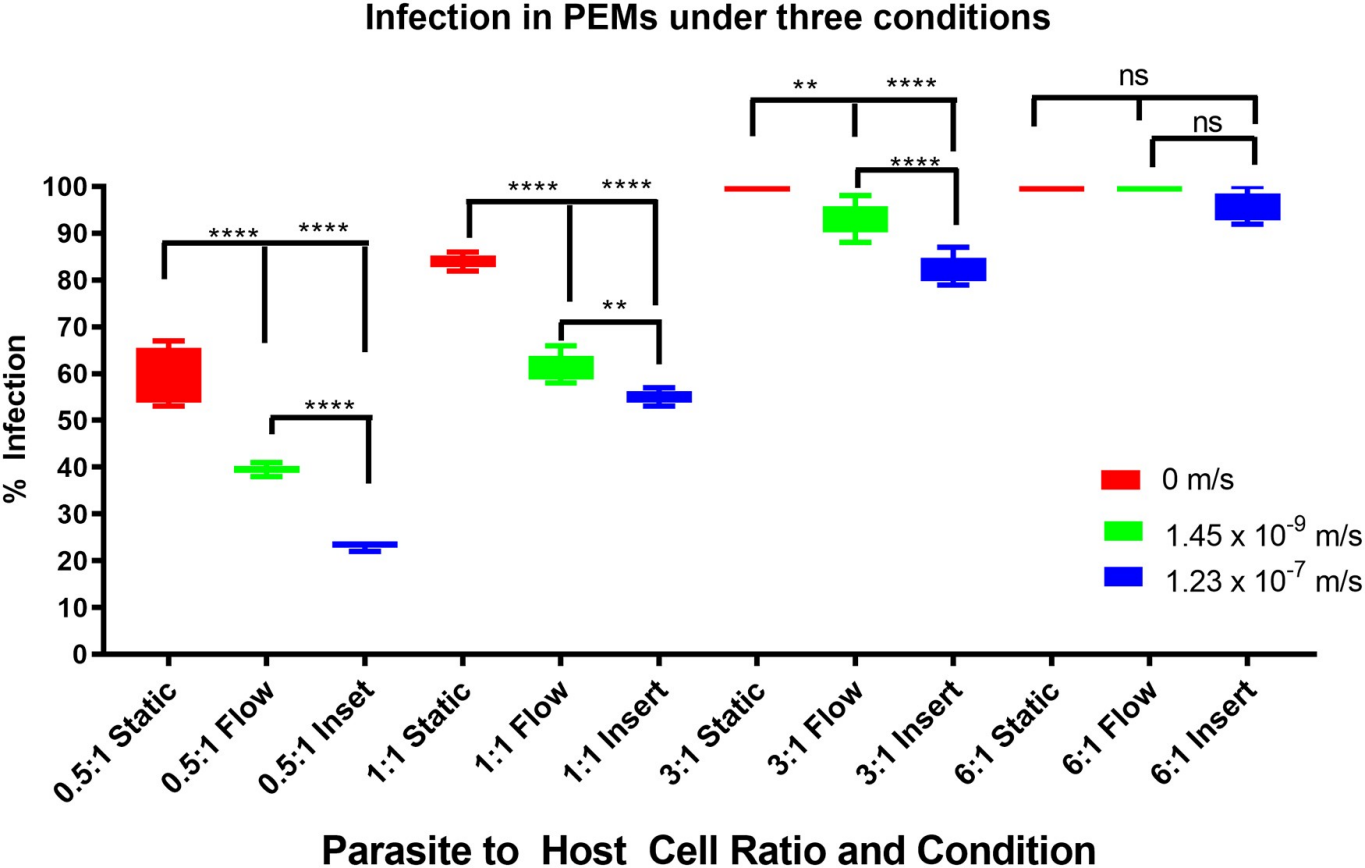
Box and whisker diagram showing the percentage of infected cells over a range of different infection ratios, of parasite: Macrophage number, and different flow conditions. Significance tested using a two tailed t-test p<0.01 = ** p<0.0001 = **** ns = not significant N = 6.

The percentage of PEMs infected after 72 hours in each of the three flow conditions i.e. static (0 m/s), base of the chamber (1.45 x 10^−9^ m/s) and on the insert (1.23 x 10^−7^ m/s) using different parasite:macrophage ratios are shown in Fig 6. The percentage infection after 24 hours, before the transfer to the media perfusion system, at each of the starting infection ratios were reproducible across all of the infected cultures at that ratio. Mean initial percentage infection levels ± SD after 24 hours were 36 ± 1, 51 ± 2, 70 ± 4 and 87 ± 5% for the four different initial infection ratios of 0.5:1, 1:1, 3:1 and 6:1 parasite concentration to cell concentration. Media perfusion was maintained over the following 72 hours.

As the flow speed of the culture medium was increased from 0 m/s in the static condition to 1.45 x 10^−9^ m/s to 1.23 x 10^-7^m/s (cells on the insert in chambers), the percentage infection of host cells decreased at all parasite to host ratios used (0.5:1, 1:1 and 3:1) (Fig 6). However, the influence of medium flow speed on macrophage infection decreased as the parasite to host ratio increases, until at a parasite to host ratio of 6:1, increasing the flow speed of the culture medium had little effect on the percentage infection levels of the host. As expected increasing the initial parasite to host ratio increases the overall infection levels after 72 hours.

Comparison of data sets showed significant differences (at least p<0.01, by one-way ANOVA) from each other except when comparing the data at the 6:1 ratio (Fig 6).

### Incorporation of 5-ethynyl-2'-deoxyuridine (EdU)

**Fig 7:**
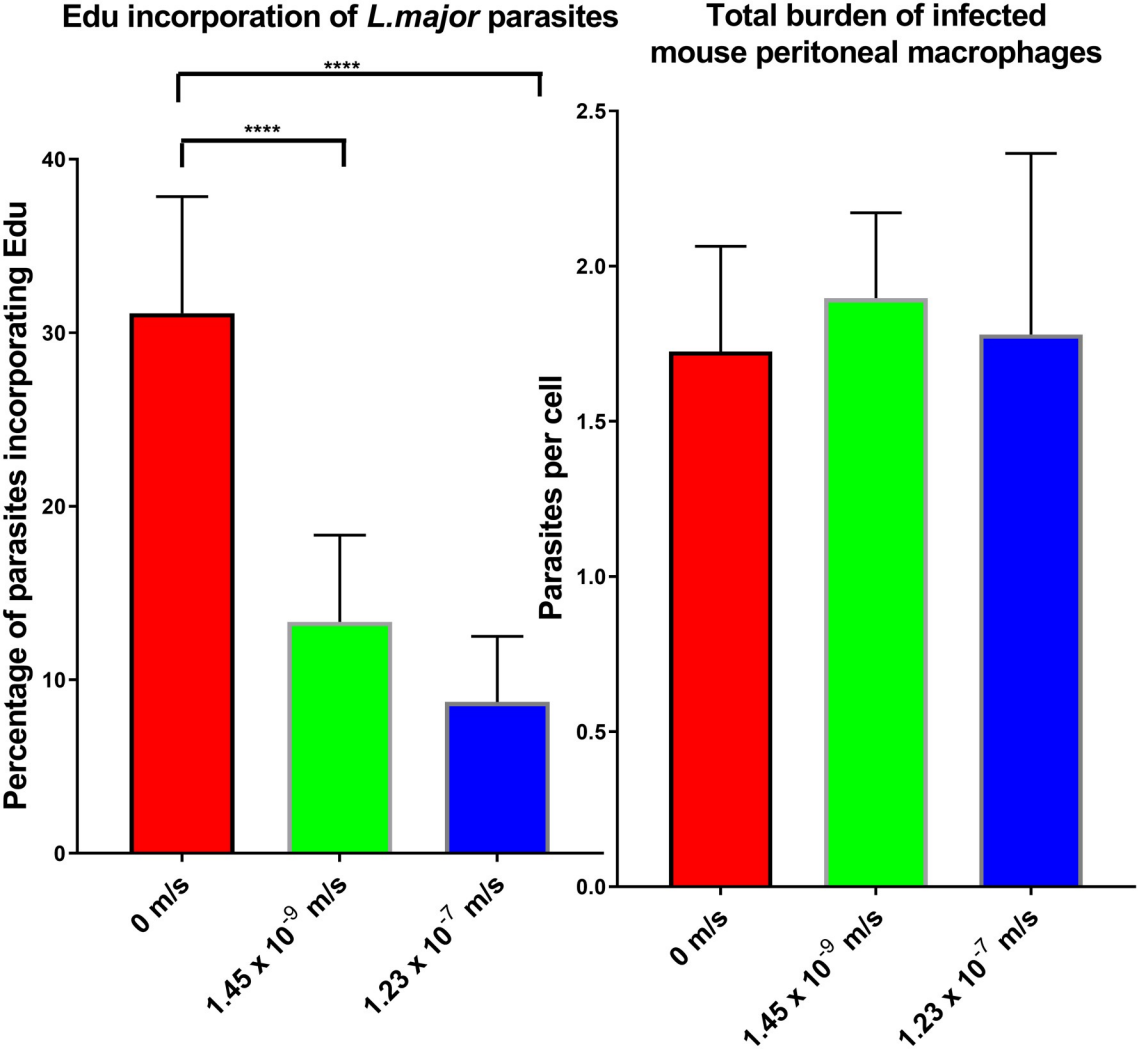
**Left: Bar graph showing percentage of *L*. *major* amastigotes that incorporated the EdU marker into DNA at the three different conditions, static (0m/s), low flow (1.45 x 10^−9^ m/s) and high flow (1.23 x 10^−7^ m/s). Right: Bar graph showing parasite burden in mouse peritoneal macrophages, at the three conditions**. * = p<0.05 N = 3.

The number of amastigotes per macrophage were counted microscopically after 24 hr under the three flow conditions, showing a similar parasite burden with approximately 2 amastigotes per infected cell (Fig 7) across the infected cells that were imaged. Percentage infection rates were identical after the first 24 hr infection (65%) regardless of the speed of media perfusion the cell would be maintained over the following 24 hr. The percentage of amastigotes that incorporated EdU into DNA was significantly lower in cultures maintained under perfusion conditions (Fig 7), with a significant reduction observed (one way ANOVA, p<0.05) in cultures in flow systems compared to static cultures after 24 hr. On average, the mean percentage of amastigotes that incorporated EdU into DNA was 31 ± 7% in cells maintained in static culture, 13 ± 5% in media flow speed of 1.45 x 10^−9^ m/s, and 9 ± 4% when media flow speed was 1.23 x 10^−7^ m/s.

### Macrophage functions

#### Phagocytosis

**Fig 8:**
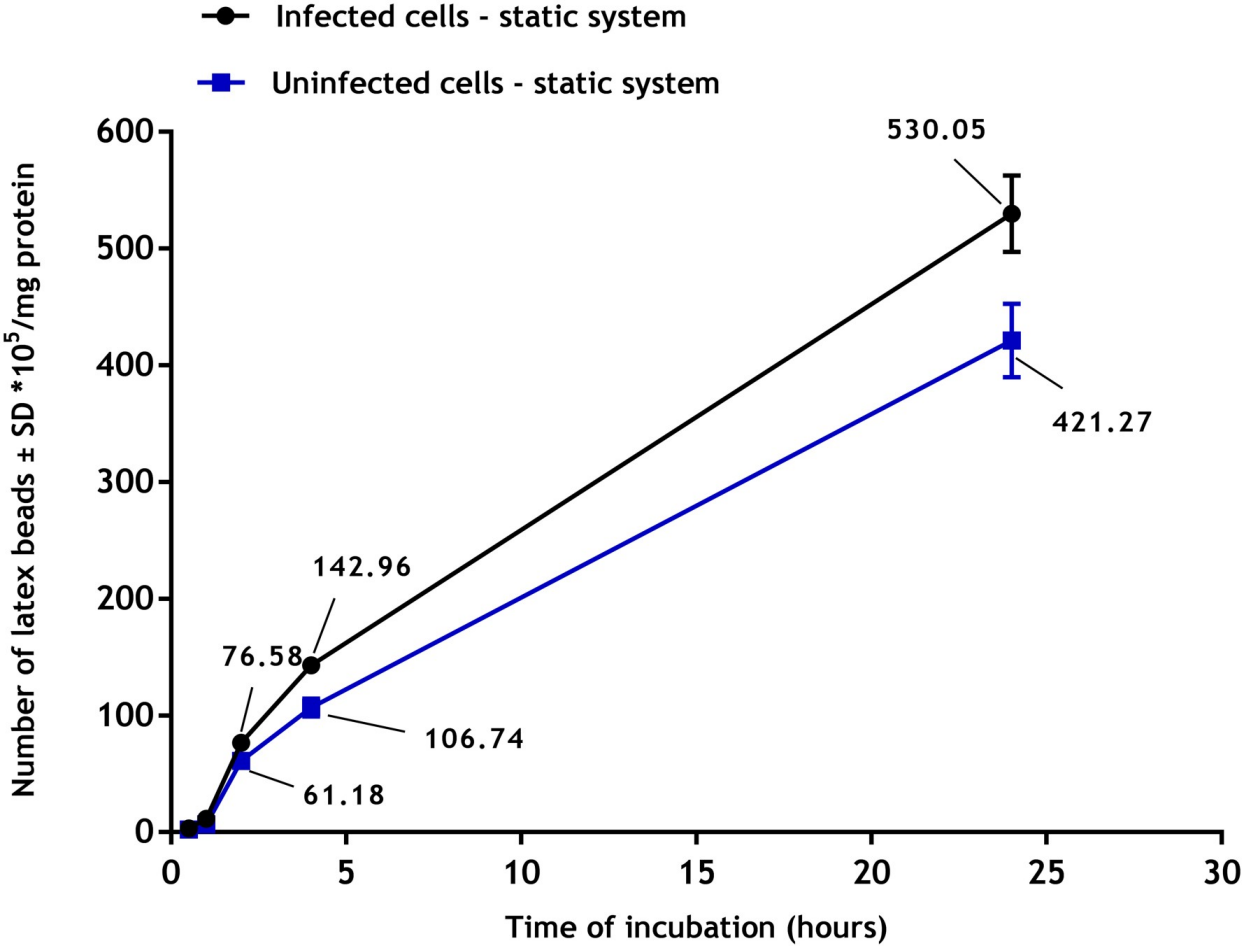
Phagocytosis of fluorescent latex beads (2 μm) by uninfected and infected PEMs in static culture system. **There is a significant increase in phagocytosis by infected PEMs compared to uninfected ones (p<0.05 by t-test)**. The data show means ± standard deviations (SD), N = 3. Infection rate was > 80%.

Phagocytosis of latex beads by uninfected and infected PEMs showed a clear time dependent response (Fig 8) with phagocytosis increasing with duration of incubation. Phagocytosis was significantly higher (p<0.05 by t-test) in infected macrophages (infection rate of > 80%) compared to uninfected ones (530± 30 x 10^5^ versus 421± 30 x 10^5^) beads/mg protein after 24 hours under static conditions.

**Fig 9:**
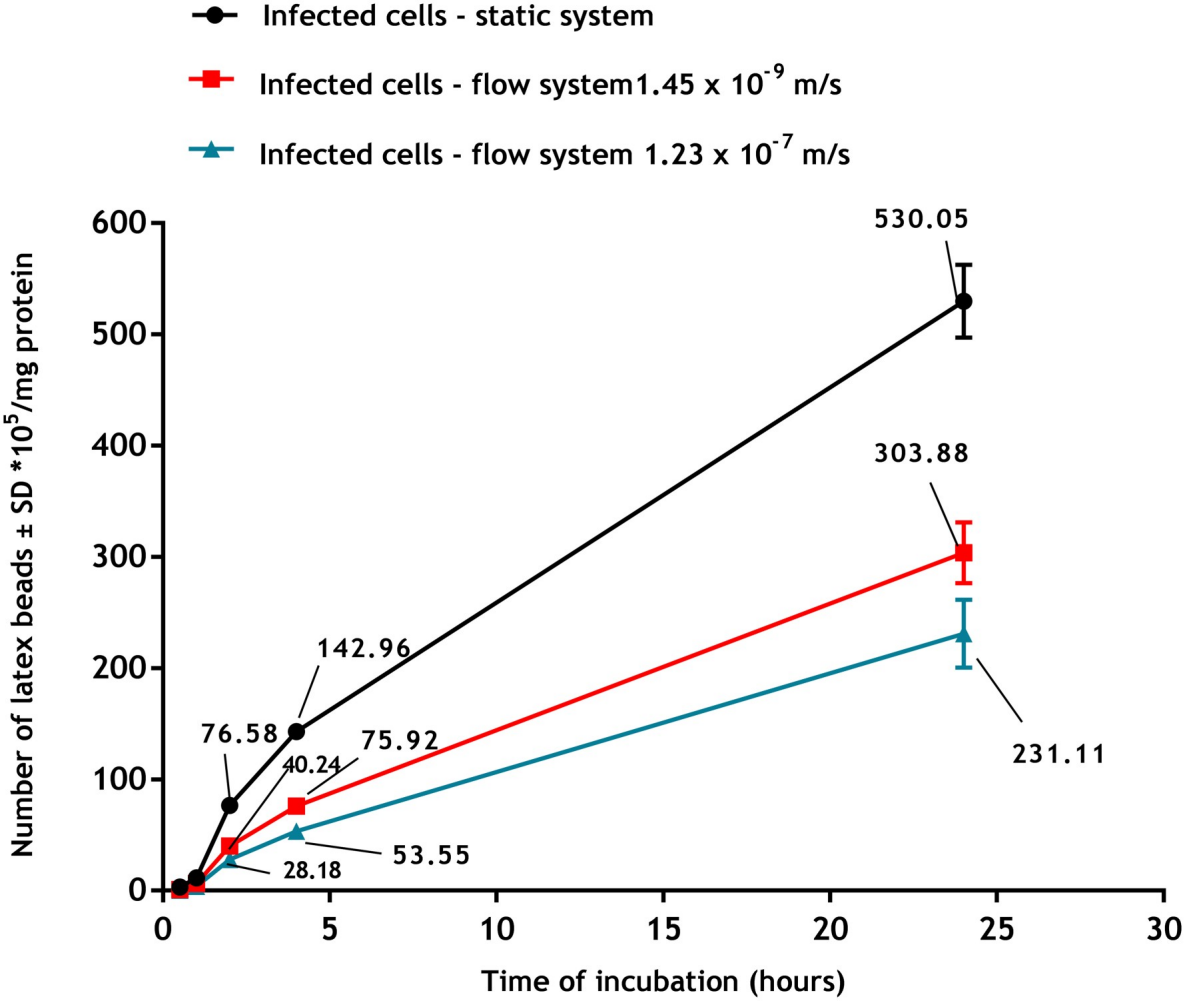
Phagocytosis of fluorescent latex beads (2 μm) by infected PEMs in the three culture systems (static, slow flow rate 1.45 x 10⁻^9^ m/s and fast flow rate 1.23 x 10^−7^ m/s). **Phagocytosis is significantly higher in static than in flow system (p<0.05 by one-way ANOVA)**. The data are means ± standard deviations (SD), N = 3. Infection rate > 80%.

Flow conditions caused a significant reduction in phagocytosis by infected macrophages as shown in Fig 9, such that after 24 h of incubation, phagocytosis had significantly decreased from 530± 30 x 10^5^ beads/mg protein in static cultures to 304± 32 x 10^5^ beads/mg protein at slow flow speed and 231± 28 x 10^5^ beads/mg protein at fast flow speed (p<0.05 by one-way ANOVA).

### Macropinocytosis

**Fig 10:**
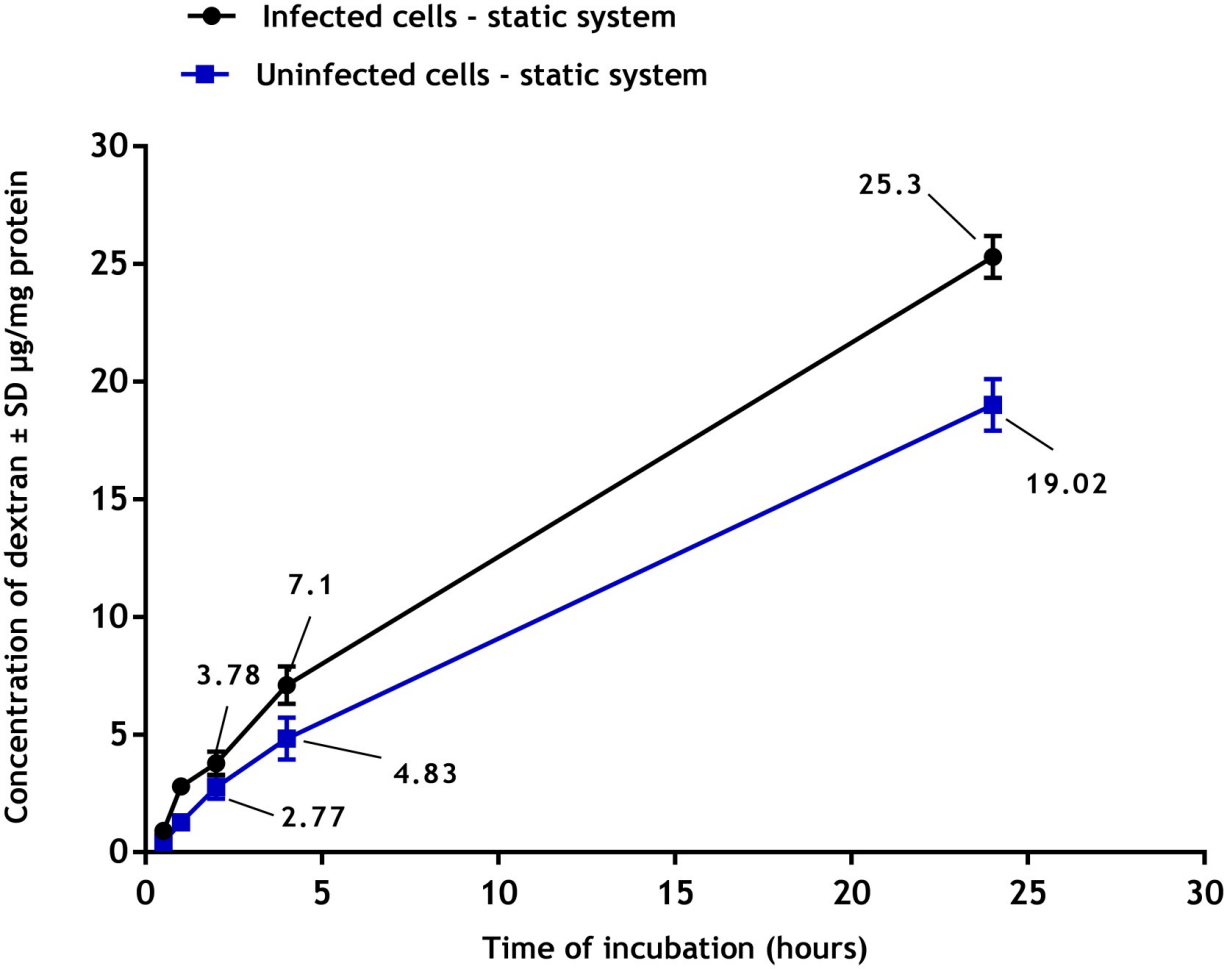
Macropinocytosis of pHrodo Red dextran by uninfected and infected PEMs in static culture system. **There is a significant increase in macropinocytosis by infected PEMs compared to uninfected ones (p<0.05 by t- test)**. The data are means ± standard deviations (SD), N = 3. Infection rate was > 80%.

Macropinocytosis of pHrodo Red dextran by uninfected and infected PEMs showed a clear time dependent response (Fig 10) with macropinocytosis increasing with duration of incubation. Macropinocytosis was significantly increased in infected PEMs (p<0.05 by t-test) compared to uninfected ones (25± 1.1 versus 19± 1.0) μg/mg protein of pHrodo Red dextran after 24 hours of incubation.

**Fig 11:**
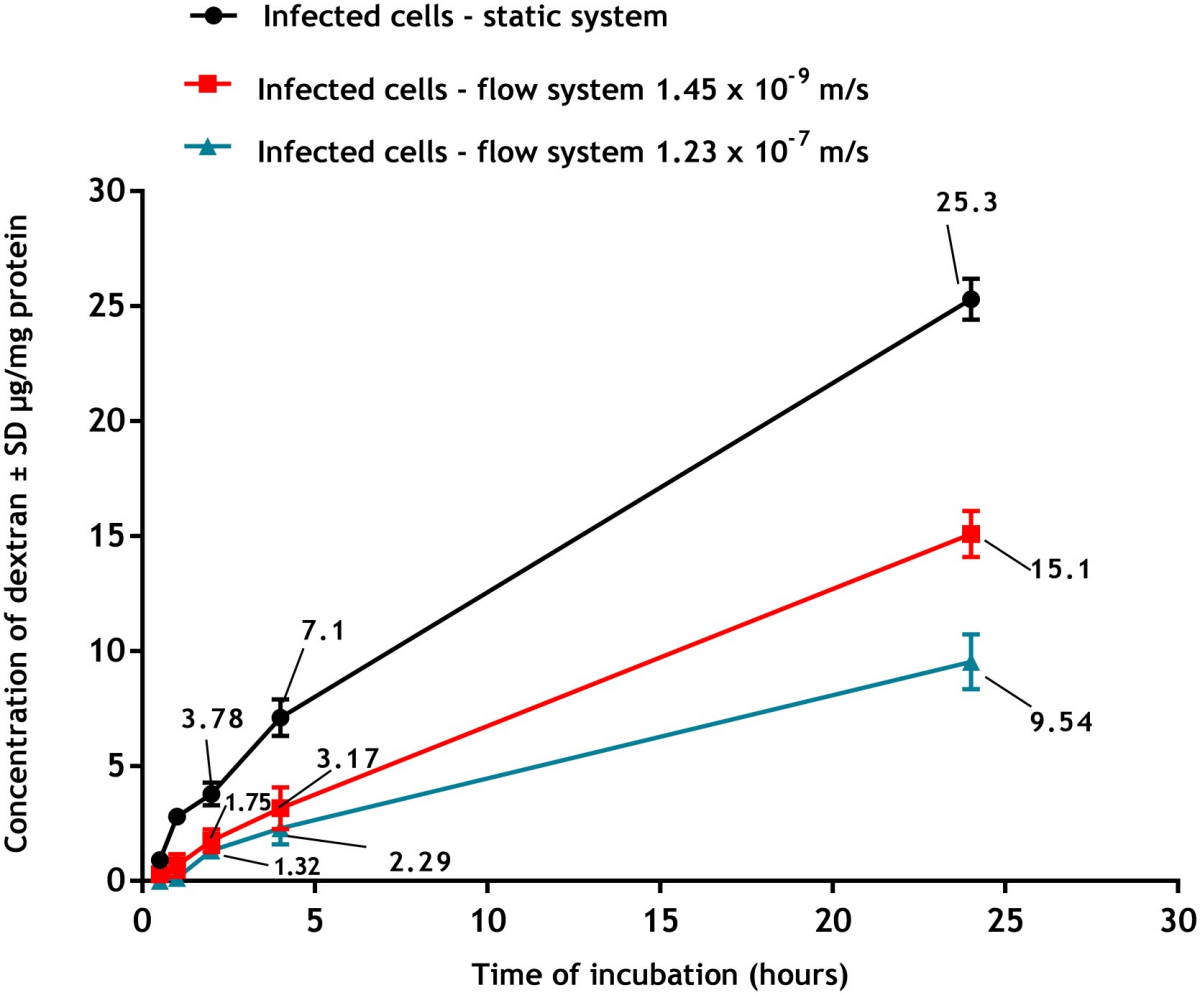
Macropinocytosis of pHrodo Red dextran by infected PEMs at the three culture systems (static, slow flow rate 1.45 x 10⁻^9^ m/s and fast flow rate 1.23 x 10⁻^7^ m/s). **Macropinocytosis is significantly higher in static than in flow systems (p<0.05 by one-way ANOVA)**. The data are means ± standard deviations (SD), N = 3. Infection rate was > 80.

Macropinocytosis was significantly reduced under flow conditions (Fig 11), with higher speed of culture medium flow causing greater reduction (p<0.05 by one-way ANOVA) so that after 24 hours of incubation with pHrodo Red dextran, macropinocytosis was 25.3± 1.1, 15.1± 0.9 and 9.5± 0.9 μg/mg protein under static, low flow and fast flow respectively.

## Discussion

### Media perfusion system and modelling

The importance of body fluid flow rates in physiology has been recognized for more than half a century [18]. Understanding the effects of fluid flow on solute transport in biological tissues and on cell-cell signalling and morphogenesis is now substantial. Media perfusion can provide more than just increased cell nourishment, it can also, for example, induce blood and lymphatic capillary morphogenesis *in vitro* [19–21], maintain the functional activity of chondrocytes and osteocytes [22–25], drive fibroblast differentiation [26] and induce cytokine production by smooth muscle cells [27]. Static systems do not offer any form of dynamic chemical or physical stimulus to cells, such as concentration gradients, flow, pressure, or mechanical stress caused by movement of fluids around them. This is a major limitation in experiments investigating cellular responses *in vitro* since the complex interplay of mechanical and biochemical factors are absent. We used the QV900 system to introduce a fluid flow component to an *in vitro L*. *major* macrophage infection model. In addition, we adapted the QV900 system to enable comparison of the effect of different flow rates to static cultures on infection of macrophages. Experiments were performed with cells cultured at the base of the chamber (‘low’ flow) and cells cultured on top of a 3D printed insert (‘high’ flow). The 3D printed insert placed into the chambers enabled us to study media flow speed at the cell surface which is in line with values reported in the literature for interstitial flow in the skin [4,6,7]. Mathematical modelling also showed that cells cultured on the 9mm insert experienced flow speeds and shear stress that were two orders of magnitude higher than those affecting cells cultured at the base of the chamber. Oxygen concentrations at the base of the chambers with the insert were also determined to be higher when compared with the chambers without the insert. The significance of our findings is that it is possible to expose cells to vastly different mechanical and chemical environments depending on where they are cultured in the chamber. This is consistent with previous studies where mathematical and computational models showed that changing the geometry of similar perfusion bioreactors has an impact on experimental conditions such as flow speed, shear stress and oxygen concentration [11].

### Infection of macrophages in the media perfusion system

The macrophage infection level caused by parasite inocula over the three different conditions varied significantly. Macrophage infection by parasites was reduced by media flow, with significant reductions seen as the media flow speed increased, as shown in Fig 5. This pattern was also seen when using a larger range of initial infection ratios (See S2 File, where we used THP1 cells as the host cells). Possible reasons for the reduction in infection rate with increasing flow rate include: (a) reduced contact time between parasites and cells, (b) increase in the supply of nutrients to the host cells, (c) effect of higher shear stress on receptors, and (d) reduced proliferation of the parasite within the host cell. Promastigotes that are external to the cells but have remained on the glass coverslip may have reduced contact with the cells [28] after transfer to the perfusion system, as they will be pushed away from the cell by the media flow. Without sustained physical contact, the parasites will not be phagocytosed and will not establish an infection within the cell^28^. A lower probability of parasite invasion into macrophages could lead to a lower infection rate, as fewer parasites would reach the phagolysosome, the site of parasite replication. The first step in the phagocytosis of the *Leishmania* promastigotes is binding to receptors on the cell surface; the Fc receptor (FcR), complement receptor type 3 (CR3), and mannose-fucose receptor have all been shown to be receptors for the parasite [29]. The flow of the media could cause a reduction in binding between the receptor and parasite. Another possible explanation for the effect of media perfusion on the final levels of infection is that the media flow provides more nutrients to the macrophages. Whilst we have considered only oxygen transport in our mathematical modelling, and demonstrated differential concentrations of oxygen at the cell surface with increasing media flow, it follows that the concentration of other important nutrients will be similarly affected. An increased supply of nutrients may provide more starting reagents for the production of anti-parasitic effectors. In addition to this beneficial effect to the host cell, it is possible that the opposite occurs in the parasite, as the parasites could expend more energy [30] resisting the flow of the media reducing successful evasion of the macrophage cellular response and replication once they have been phagocytosed. Cells are sensitive to shear stress and change behaviour depending on physical forces [31,32]. They have been shown to respond to shear stress by changing shape [33], phenotype [34], and release of proteins/chemicals [35]. This stress will undoubtedly have an impact on the phagocytosis process [36].

Another possibility for the lower percentage infection in media perfusion system maintained cultures could be that the rate of parasite proliferation in the host cell is altered. The EdU incorporation assay demonstrated that fewer parasites are actively incorporating the labelled DNA base under media perfusion conditions. Although the average amastigote burden of the PEMs was the same under static, low flow and high flow, the lower EdU incorporation at higher flow speeds shows that parasites were replicating to a lower extent. This is a phenomenon also seen in plankton where increased flow reduces biomass build up [37].

Flow also affected phagocytosis and macropinocytosis of macrophages. Firstly, we established there was a significant increase in both cell functions in PEMs infected with *L*. *major* compared to uninfected cells. These data are consistent with results described elsewhere, for example macrophages infected with either *L*. *donovani* or *L*. *mexicana* increased their pinocytic rates as measured by a fluorescent probe (fluorescein isothiocyanate dextran) [38]. Similar observations have been reported with RAW 264.7 macrophages infected with *L*. *major* showing increased uptake of fluorescently labelled liposomes [38]. This might be due to morphological changes of the infected cells or the parasitic infection may alter both the metabolic activity of the macrophages and their ability to ingest particulate material [39]. Our results demonstrated that phagocytosis and macropinocytosis were significantly decreased by media flow and that increasing the media flow speed caused a further reduction in the uptake. This is consistent with previous reports of decreased uptake of fluorescein isothiocyanate (FITC) -poly (ethylene glycol) diacrylate particles (200 nm diameter) by human umbilical vein endothelial cells in a dynamic cell culture system exposed to shear stress of 10 dynes/cm^2^ compared to the static culture [40]. Similar findings were also seen with a lower cellular uptake of solid silica particles (350 nm) by RAW 264.7 macrophages under dynamic condition compared with the static culture [41]. One explanation given was that the static system conditions might cause sedimentation of the beads on the cell surface or exposure to higher concentrations of pHrodo Red dextran resulting in a local increase in their concentrations [42]. In contrast, medium flow prevents such localization of materials with subsequently reduced uptake [43].

In conclusion, in the media perfusion *Leishmania*–macrophage model flow speed was shown to affect infection rate even at interstitial fluid rates. This could have an impact on the development of *Leishmania* infection in skin especially when considering the possible higher flow rates in inflammatory sites. The role of mathematical modelling was essential to understanding different chemical and physical conditions resulting from the flow and, highlighting the need for mathematical modelling to be further integrated into this approach. The collateral effects of flow on pathogen replication rate and on host cell metabolism, as indicated by reduction in phagocytosis and macropinocytosis, further indicates research avenues and how these models might be used in studies on immune responses and drug and vaccine discovery. Additionally, our combined experimental and modelling approach has allowed us to generate hypotheses which we will test in future through the development of more advanced mathematical models and experiments.

## Supporting information

S1 File Supplementary material for “Development of a media perfusion model of macrophage infection by *Leishmania major*”. (DOCX)

S2 File Supplementary material 2 for “Development of a media perfusion model of macrophage infection by *Leishmania major*”. (DOCX)
