# Supplementary material for: Development of an in vitro media perfusion model of Leishmania major macrophage infection
Source: PLoS One. 2019 Jul 24;14(7):e0219985. doi: 10.1371/journal.pone.0219985 (PMC6656416; doi:10.1371/journal.pone.0219985)
Supplement: S1 File — (DOCX) [file pone.0219985.s001.docx]

**Supplementary material 1 for “Development of a media perfusion model of macrophage infection by Leishmania major”**

**Fluid dynamics in the chambers**

Assuming the medium is an incompressible Newtonian fluid, the flow velocity and pressure are described by the continuity and Navier-Stokes equations:

$$\boldsymbol{\nabla}\boldsymbol{\cdot u}=0,$$

$$\rho\frac{\partial\boldsymbol{u}}{\partial t}+\rho\left( \boldsymbol{u}\cdot\nabla\right)\boldsymbol{u}= -\nabla p+ \mu\nabla^{2}\boldsymbol{u},$$

where $\boldsymbol{u}$ (m s^-1^) is the velocity field, $\rho$ (kg m^-3^) is the fluid density, $p$ (Pa) is the pressure and $\mu$ (Pa s) is the dynamic viscosity. For simplicity, the values of $\rho$ and $\mu$ are chosen under the assumption that the fluid is water, but it is recognised that these parameters may vary depending on the specific fluid used. Initially, the fluid velocity is zero in the chamber. At the inlet, we assume a parabolic velocity profile derived from the flow rate and at the outlet, we assume zero pressure. No slip and no penetration conditions are imposed on the outer walls of the chambers and connecting tubes.

**Oxygen transport in the chambers**

The transport of oxygen through the fluid is described by a convection-diffusion equation:

$$\frac{\partial c}{\partial t}+\left( \boldsymbol{u}\cdot\nabla\right)c=D\nabla^{2}c,$$

where $c$ (mol m^-3^) is the concentration of oxygen and $D$ (m^2^ s^-1^) is the diffusion coefficient of oxygen in water. Initially, the oxygen concentration is zero in the chamber. We assume a constant supply of oxygen of concentration $c_{in}$ at the inlet and a convective flux at the outlet. We assume that the chambers and connecting tubes are impermeable to oxygen and represent this by a zero flux condition on all outer walls.

The consumption of oxygen by the cells is described by Michaelis-Menten kinetics and we implement this through the following flux boundary condition at the base of the chamber:

$$\boldsymbol{n}\cdot\left( -D\nabla c+ \boldsymbol{u}c \right)= \frac{{dV}_{max}c}{K_{m}+c} ,$$

where $\boldsymbol{n}$ is an outward facing normal, $d$ (cell m^-2^) is the cell density, ­$V_{max}$ (mol cell^-1^ s^-1^) is the maximum oxygen consumption rate and $K_{m}$ (mol m^-3^) is the Michaelis-Menten constant.

**Parameter values**

The table below shows the parameter values that were used in the simulations.

| Parameter | Description | Value | Reference |
| --- | --- | --- | --- |
| $\rho$ | Fluid density | 9.95 x 10^2^ kg m^-3^ | 1 |
| $\mu$ | Fluid dynamic viscosity | 7.32 x 10^-4^ Pa s | 1 |
| $D$ | Oxygen diffusion coefficient in water | 3.00 x 10^-9^ m^2^ s^-1^ | 2 |
| $c_{in}$ | Inlet oxygen concentration | 0.21 mol m^-3^ | 2 |
| $d$ | Cell density (number of cells/area of cells) | 3.54 x 10^9^ cell m^-2^ | this study |
| $V_{max}$ | Maximum oxygen consumption rate | 1.00 x 10^-18^ mol cell^-1^ s^-1^ | 3 |
| $K_{m}$ | Michaelis-Menten constant | 6.30 x 10^-3^ mol m^-3^ | 4 |

S1 Table. Parameter values used in the simulations.

**Implementation**

COMSOL Multiphysics, a commercially available finite element analysis software, was used to perform the simulations in this study. The built-in ‘finer’ mesh setting was used since reducing the mesh setting to ‘extremely fine’ altered the results by less than 1%. For the purposes of this study, only the steady-state solution was considered, since steady-state was assumed to be achieved rapidly in the experiments. Simulations were performed firstly for a single 3D chamber geometry and subsequently for six 3D chambers connected in series, with the first three chambers having cells residing at the base and the last three chambers having cells raised 9mm.

**References**

1. Crittenden JC, Trussell RR, Hand DW, Howe KJ, Tchobanoglous G. MWH’s Water Treatment: Principles and Design, Third Edition. John Wiley & Sons. 2012.
2. Mazzei D, Guzzardi MA, Giusti S, Ahluwalia A. A low shear stress modular bioreactor for connected cell culture under high flow rates. Biotechnology and Bioengineering. 2010 May 1;106(1):127-37.
3. Yakh’ev AV, Osipov A, Azizova OA, Korbina LG, Velichkovskii BT, Vladimirov YA. Oxygen consumption by peritoneal macrophages measured by electron paramagnetic resonance. Bulletin of Experimental Biology and Medicine. 2004; 99:464-466.
4. Weise F, Fernekorn U, Hampl J, Klett M, and Schober A. Analysis and comparison of oxygen consumption of HepG2 cells in a monolayer and three-dimensional high density cell culture by use of a matrigrid. Biotechnology and Bioengineering. 2013 Sep;110(9):2504-2512.
