# Supplementary material for: Development of an in vitro media perfusion model of Leishmania major macrophage infection
Source: PLoS One. 2019 Jul 24;14(7):e0219985. doi: 10.1371/journal.pone.0219985 (PMC6656416; doi:10.1371/journal.pone.0219985)
Supplement: S2 File — (DOCX) [file pone.0219985.s002.docx]

**Supplementary material 2 for “Development of a media perfusion model of macrophage infection by *Leishmania* major”**

Data used to create figure 6 of the main manuscript was drawn from a number of different experimental data sets. All data shown in this supplementary material is conducted using the methods described in the manuscripts materials and methods section but use THP1 cells (ATCC® TIB-202™, UK) instead of peritoneal macrophages.

**Static and Flow at 50ul/min**

S2 Fig 1. Box and whisker diagram showing the percentage of infected cells over a range of different infection ratios, of parasite: macrophage number, and either no flow or flow at a rate of 50ul/min. Significance tested using a two tailed t-test p<0.01 = ** p<0.0001 = **** N=9

**Static, Flow 360ul/min and Flow with insert 360ul/ml**

S2 Fig 2. Box and whisker diagram showing the percentage of infected cells over a range of different infection ratios, of parasite: macrophage number, and a range of flow conditions, including the use of an insert. Significance tested using a two tailed t-test p<0.01 = ** p<0.0001 = **** N=3

**Static and Flow 1000ul/min**

S2 Fig 3. Box and whisker diagram showing the percentage of infected cells over a range of different infection ratios, of parasite: macrophage number, and either no flow or flow at a rate of 1000ul/min. Significance tested using a two tailed t-test p<0.01 = ** p<0.0001 = **** N=6

Whilst the data used to create Fig 7 was mostly formed of manually counting infected macrophages using a confocal microscope (Zeiss LSM510 Axiovert, Germany). Some example images of the Edu incorporation assay were captured.


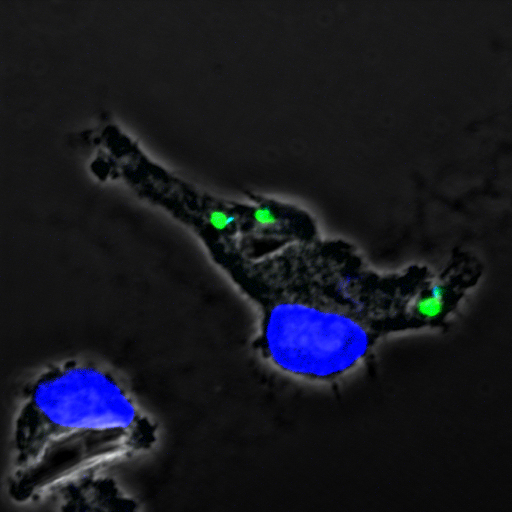

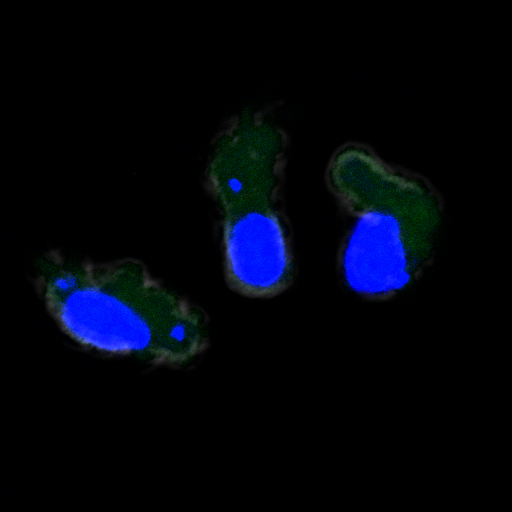


S2 Fig 4. Example 2D images of macrophages infected with by *Leishmania* major as part of the Edu incorporation assay taken on a confocal microscope (Zeiss LSM510 Axiovert, Germany). Right – Macrophage maintained under static conditions showing Edu incorporation of dividing parasites (Green). Left – Macrophages maintained under flow conditions (1.23 x 10^-7^ m/s) showing infection of the macrophage but non dividing parasites (Blue).
